# Expression and Prognostic Significance of Metastasis-Associated Protein 1 in Gastrointestinal Cancer

**DOI:** 10.3389/fonc.2020.542330

**Published:** 2020-12-21

**Authors:** Pengping Li, Wei Cao, Rui Ding, Mengqiu Cheng, Xin Xu, Sihan Chen, Bo Chen, Guodong Cao, Maoming Xiong

**Affiliations:** ^1^ Department of General Surgery, First Affiliated Hospital of Anhui Medical University, Hefei, China; ^2^ Department of General Surgery, First People’s Hospital of Xiaoshan, Hangzhou, China; ^3^ Department of Emergency, the Lu’an Affiliated Hospital of Anhui Medical University, Lu’an, China

**Keywords:** gastrointestinal cancer, metastasis-associated protein 1 (MTA1), prognosis, meta-analysis, immunohistochemistry

## Abstract

**Background:**

Metastasis-associated protein 1 (MTA1) has been considered as a transcriptional regulator, which is significantly related to the prognosis in various types of tumors. However, whether MTA1 is a potential prognostic index of gastrointestinal cancer (GIC) remains controversial. The current meta-analysis was performed to evaluate the role of MTA1 expression in the prediction of the clinicopathological features and survival in GIC cases. And the results of gastric cancer were verified by immunohistochemistry (IHC).

**Methods:**

Eligible studies assessing the relationship between MTA1 and GIC by IHC were searched in the PubMed, Cochrane, Ovid, Web of Science and CNKI databases by various search strategies. The STATA 16.0 software was applied to gather data and to analyze the potential relationship between MTA1 and GIC. The expression level of MTA1 was examined in 80 GC samples by IHC assay. SPSS 20.0 was applied for statistical analysis, and the survival curves were calculated by the Kaplan-Meier method. The data of 95% CI was displayed as “[a-b]”.

**Results:**

According to the meta-analysis, the expression level of MTA1 was tightly associated with the tumor size (OR=1.82 [1.16–2.84], *P*=0.009), tumor tissue differentiation (OR=1.71 [1.24–2.37], *P*=0.001), depth of invasion (OR=3.12 [2.55–3.83], *P<*0.001), lymphatic metastasis (OR=2.99 [2.02–4.43], *P*<0.001), distant metastasis (OR=4.66 [1.13–19.24], *P=*0.034), TNM stage (OR=4.28 [2.76–6.63], *P*<0.001). In addition, MTA1 played the negative effects in 1- (RR=2.48 [1.45–4.25], *P*=0.001), 3- (RR=1.66 [1.30–2.11], *P*<0.001) and 5-year (RR=1.73 [1.37–2.20], *P*<0.001). Study in subgroup, grouped by language and tumor type, we reached similar conclusions. Further validation by IHC yielded similar conclusions. Tumor size (*P*=0.008), lymph node metastasis (*P*=0.007) and distant metastasis (*P*=0.023) significantly accompanied with higher expression of MAT1 in GC cases. Besides, the expression level of MTA1 was statistically significantly correlated with OS in GC cases (HR=2.061 [1.066–3.986], *P*=0.032), which suggested that MTA1 might be an independent prognostic marker for GC. Finally, we verified the correlation between the expression level of MTA1 and prognosis of GC in 80 GC samples.

**Conclusions:**

MTA1 is tightly associated with metastasis-related factors and may constitute a promising prognostic factor of GIC.

## Background

Gastrointestinal cancer (GIC) is a group of malignant tumors, including esophageal squamous cell carcinoma (ESCC), gastric cancer (GC) and colorectal cancer (CRC), which account for more than 27% of the total newly diagnosed tumors worldwide, and account for about 37% of tumor-related death events among all types of tumors (1, 2). ESCC is one of the most common tumors in the world, and its 5-year overall survival rate is less than 30% (3, 4). Gastric cancer (GC) is also one of the most common malignant tumors, the incidence of which at the 6th position reported by the “2012 Global Cancer Statistics” (1). In Europe, CRC is the second most common malignant tumor and the second most common cause of cancer death (5). As we all know, the occult distant metastasis of gastrointestinal tumors is one of the important reasons for tumor recurrence post-surgical resection, as well as one of the important reasons for the low quality of life and high mortality of tumor patients. Nevertheless, current assessment methods for tumor prognosis cannot meet our needs, to find out the patients who may have a high risk of tumor recurrent and disease progression. Therefore, the work of finding the new biomarkers for gastrointestinal tumor prognosis prediction is necessary.

GIC is always associated with a high risk of metastasis, with its occurrence accompanied by lymphatic metastases and distant metastases. The evaluations of lymph node, distant metastasis and tumor size are taken into consideration both in clinical and pathological diseases staging, among which the first two indexes are considered as the pivotal indicators for predicting clinical outcomes. Nevertheless, developed evidences imply that current staging criteria are weakening in differentiating the prognostic features of GIC cases. Metastasis is not a one-step process, which contains the dissemination of primary cancer cells into surrounding tissues and cycle system, and colonization at distant metastatic sites (6). These contribute to morbidity and mortality in cancer cases. So, further understanding the bimolecular procedure of metastasis and developing new prevention strategies may improve clinical treatment effect.

Various kinds of factors (miRNA, LncR NA, DNA and protein) are associated with prognosis in cancer, some of which are involved in the regulation of cancer invasion and metastasis. Metastasis-associated proteins (MTAs) playing prominent roles in that, especially MTA1. MTAs are consisting of MTA1, MTA2, and MTA3, all of which can directly bind with the nucleosome remodeling and histone deacetylation (NuRD) complex, which plays a transcriptional regulatory role *via* histone deacetylation and chromatin remodeling (7). MTA1 is firstly found at a higher level in metastatic rat breast adenocarcinoma cell lines as compared with poorly metastatic counterparts, and is considered a tumor invasion and metastasis-related gene for its over-expression is positively associated with tumor invasion and metastasis (8). Toh et al. (9) demonstrated that the higher expression level of mRNA of MTA1 was tightly associated with the depth of tumor invasion and lymphatic metastasis, especially for lymph node metastasis. Song et al. (10) pointed out that the expression level of MTA1 was an independent risk factor in cancer prognosis. Indeed, a higher expression level of MTA1 accompanies with worse disease-free survival and 5-year survival rate in cancer patients.

To our knowledge that few reports summarized the prognostic significance of MTA1 in solid tumors by meta-analysis, and the role of MTA1 in the evaluation of GIC prognosis remains inconclusive and unclear. Thus, this meta-analysis was a necessity to assess the MTA1 expression for patterns and associations with prognosis and survival in patients with GIC. Similar results were also obtained through further validation by IHC.

## Methods

### Search Strategy

The method of this meta-analysis refers to an article published before (11), and the specific analysis process is as follows. Relevant articles were searched by two researchers independently in the PubMed, Cochrane, Ovid, Web of Science and CNKI databases, from inception to Oct 2020.The search strategies included (“MTA1” OR “Metastasis-associated protein 1”) AND (“esophagus” OR “esophageal” OR “esophagus” OR “gastric” OR “stomach” OR “cardia” OR “colon” OR “colorectal” OR “gastrointestinal” OR “sigmoid” OR “sigmoidal” OR “rectal” OR “anal” OR “rectum” OR “digestive tract”) AND (“carcinoma” OR “cancer” OR “tumor” OR “neoplasm” OR “tumor” OR “malignancy”). Full texts were reviewed to assess whether the reports met the inclusion criteria.

### Inclusion and Exclusion Criteria

Studies were considered to be relevant if they met the following criteria (1): GIC diagnosis (2); immunohistochemistry (IHC) as evaluation method (3); association of MTA1 with GIC assessed (4); English and Chinese as publication language. Exclusion criteria were (1): data repetition (2); reviews (3); case reports (4); evaluation method not IHC (5); erroneous data.

### Data Extraction and Quality Evaluation

According to the above selection criteria, all selected data in each study were rigorously extracted independently by two researchers (Pengping Li and Wei Cao). All disagreement has got consensus by a team discussion. The extracted data included 1st author’s name, the time of publication, count of patient, clinical and pathological parameters, and survival status. Two investigators independently evaluated the quality of eligible studies by the Newcastle- Ottawa scale (12).

### Gastric Cancer Sample Preparation

The whole work in this paper was approved by The No.1 Affiliated Hospital of Anhui Medical University (AMU) Review Board and the Ethics Committees of AMU. Eighty matched gastric cancer paraffin-embedded sections and three paired fresh-frozen samples of gastric cancer were collected. All samples came from the patients, whoever got gastrectomy at the No.1 Affiliated Hospital of AMU from 2013 to 2015. Each of the above gastric cancer samples got a consistent pathological diagnosis by at least two pathologists. Clinical outcome was evaluated and recorded from the surgical treatment day to that of an event (i.e. patient death or tumor recurrence) or withdrawal.

### Immunohistochemistry

Simply put, as in our previous work. GC tissues were washed by pre-cooling phosphate-buffered saline (PBS) (1X) to remove the blood and any other impurities. Then these issues were fixed in formalin (10%) for 30 min and followed by being embedded in paraffin for making eligible sections. Next, these sections were de-paraffinized and hydrated by xylene and serially diluted ethanol under the protocol. The endogenous peroxidase was inactivated by being treated with H_2_O_2_ (3%) for 10 min. Following, a citrate solution was applied for antigen retrieval with a microwave oven. And then, these sections were incubated with the required primary antibody for an appropriate time (12h to 16h) at 4°C. Then, these sections were treated with three 5-min mild washing in PBS (1X), followed by 20-min-treatment of secondary antibody. Finally, diaminobenzidine tetrahydrochloride (DAB) was applied before counterstaining with hematoxylin.

### Evaluation of Immunohistochemistry

Two independent pathologists evaluated the expression intensity of MTA1 in IHC staining sections, basing on a semiquantitative grading system (includes the proportion of stained cells and the staining intensity). Staining intensity was scored in four degrees as: 0 (negative,-), 1 (weak, +), 2 (moderate, ++), and 3 (strong, +++). The proportion of positive epithelial cells was scored in four degrees as: 0 (no staining, -), 1(<1/3 staining, +), 2 (1/3 to 2/3 staining, ++), and 3 (>2/3 staining, +++). And the histological score was performed basing on the above results. Finally, 3–4 score is defined as positive expression, while the 0–2 score is defined as a negative expression.

### Statistical Analysis

In this paper, the meta-analysis was carried out with the help of STATA software (version 16.0, StataCorp LP, College Station, TX, USA). Two parameters as Crude odds ratios (ORs) and 95% conﬁdence intervals (CIs) were applied to evaluate the strength of the correlation between the expression level of MTA1 and clinic-pathological indexes in GC cases. Both risk ratios (RRs) and 95% CIs were carried out in estimating the potential correlation between MTA1 status and overall survival (OS). The *I* ^2^ index was applied to assess statistical heterogeneity across the studies. A random-effects model was applied in case with significant heterogeneity (*I* ^2^>50% or *P*<0.1); otherwise, a fixed-effects model was employed. To estimate the potential publication bias, Begg’s rank correlation test and Egger’s weighted regression method were applied, with *P*<0.05 indicating statistically significant publication bias.

SPSS 16.0 (USA) was applied for any other statistical analysis. All experimental data are displayed as the mean ± SD. Chi-square (χ^2^) test was also performed for the data without normal distribution. Survival curves were performed by the Kaplan–Meier method, and the statistical significance was evaluated *via* the log-rank test. Statistical significance was defined as *P*< 0.05.

## Results

### Research Characteristics

**Figure 1:**
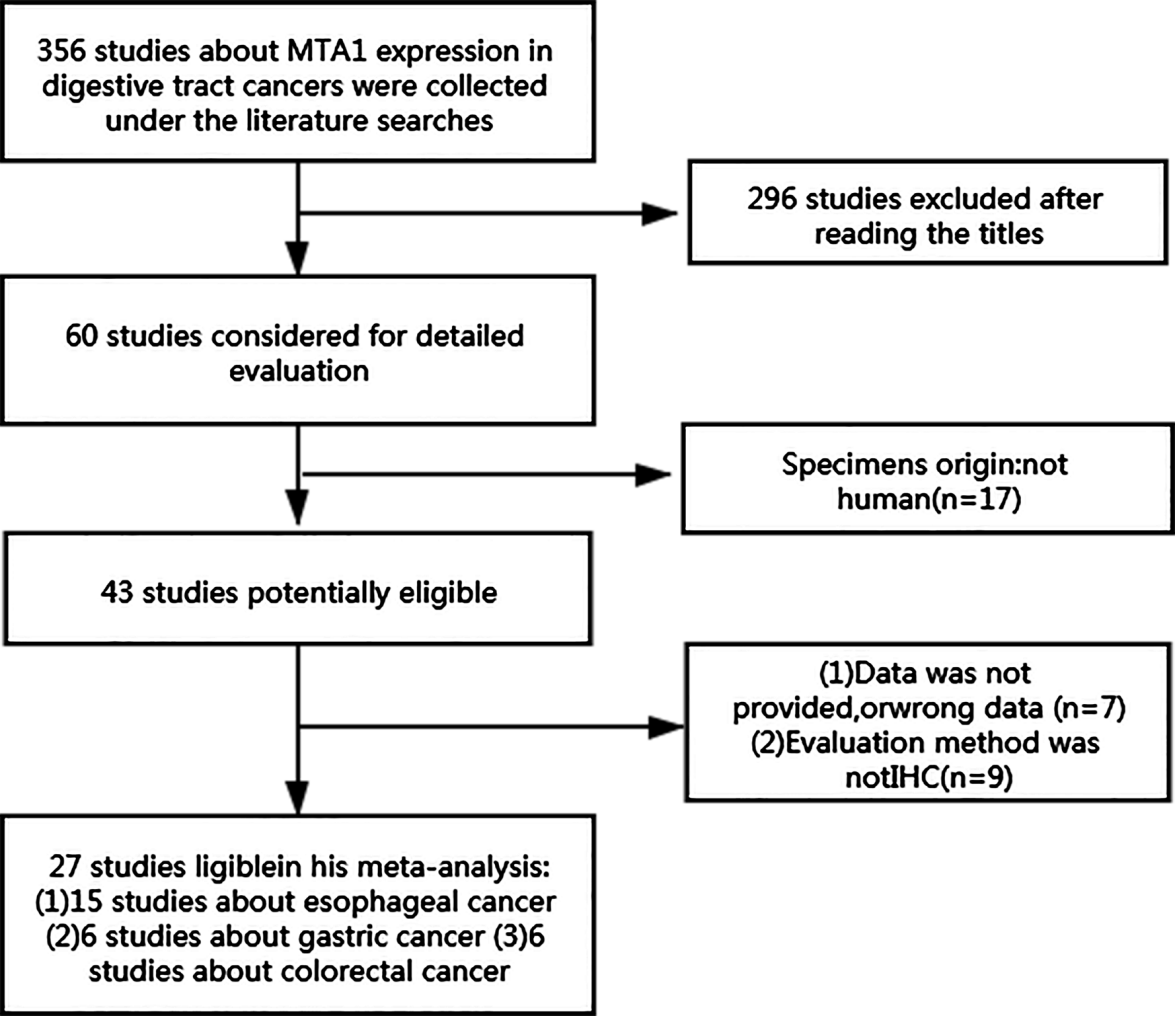
Flow diagram of the study selection procedure.

The results of searching contained 356 eligible studies that can evaluate the potential relationship between the expression level of MTA1 and disease characteristics of GICs. After primary evaluation with titles, 60 reports were retained. Then, according to the description in abstracts and full texts, we found 15 studies assessed MTA1 and ESCC (10, 13–26), 6 evaluated MTA1 and GC (27–32), and 6 analyzed MTA1 and CRC (33–38), and all of which met the inclusion criteria (**Figure 1**). The included studies are described in detail in **Supplementary Table 1**. Antibodies (used for IHC) and assessment methods (MTA1 expression in IHC) in the eligible studies are displayed in **Supplementary Table 2**. MTA1 expression in 2,952 GIC patients was determined, and the sample size ranged from 44 to 436 in the 27 included reports. All of the above eligible studies applied IHC biotechnology to explore the relationship between the MTA1 of GIC. The overall positive expression rate of MTA1 in GIC samples was 57.5% (1,490/2,952), in which 51.8% (798/1,541) of ESCC samples with over-expression of MTA1, 43.8% (389/889) of GC specimens with over-expression of MTA1, and 58.0% (303/522) of CRC samples with over-expression of MTA1.

### Associations of MTA1 Expression With Clinicopathological Parameters in Gastrointestinal Cancer Patients

**Figure 2:**
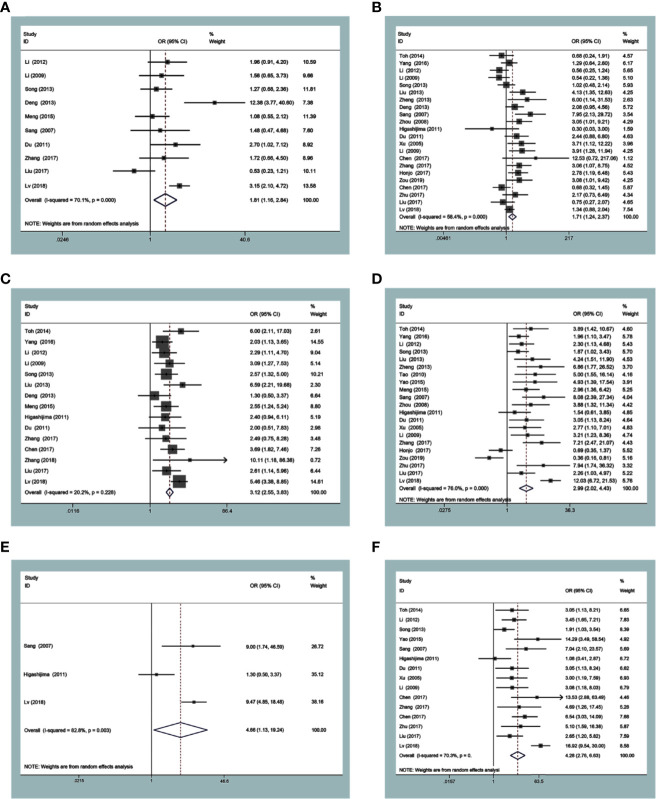
Forest plots of odds ratios for associations of metastasis-associated protein 1 (MTA1) with tumor size **(A)** and differentiation **(B)**, depth of invasion **(C)**, lymph node metastasis **(D)**, distant metastasis **(E)** and TNM stage **(F)**.

The expression of MTA1 was statistically significantly related to some metastasis-associated clinical indexes. As displayed in **Table 1**, over-expression of MTA1 was close to tumor size (OR=1.82 [1.16–2.84], *P*=0.009, **Figure 2A**), tumor tissue differentiation (OR=1.71 [1.24–2.37], *P*=0.001, **Figure 2B**), depth of invasion (OR=3.12 [2.55–3.83], *P<*0.001, **Figure 2C**), lymph node metastasis (OR=2.99 [2.02–4.43], *P*<0.001, **Figure 2D**), distant metastasis (OR=4.66 [1.13–19.24], *P=*0.034, **Figure 2E**) and TNM stage (OR=4.28 [2.76–6.63], *P*<0.001, **Figure 2F**). These results demonstrated that the higher expression level of MTA1 conferred the higher risk of digestive tract wall invasion, lymphatic metastasis and distant metastasis, which lead to advancing TNM stage. However, other clinicopathological variables had no associations with MTA1 expression, including gender, age, and vascular invasion.

**Table 1:** Meta-analysis of a putative association between clinicopathological parameters and MTA1 expression in gastrointestinal cancer.

Parameters	Number of studies	Number of patients	Heterogeneity	Model	OR(95%CI)	*P* value
			*I* ^2^ (%) *P* value			
Sex (male/female)	24	2,751	22 0.17	FE	0.91(0.77, 1.08)	0.279
Age (>60/<60)	10	960	0 0.94	FE	0.83(0.64, 1.10)	0.184
Tumor size (>5cm/<5cm)	10	1,428	70 0	RE	1.82(1.16, 2.84)	**0.009**
Differentiation (poor/well)	22	2,550	58 0	RE	1.71(1.24, 2.37)	**0.001**
Depth of invasion (T3+T4/T1+T2)	15	1,988	20 0.23	FE	3.12(2.55, 3.83)	**<0.001**
LN metastasis (positive/negative)	21	2,386	76 0	RE	2.99(2.02, 4.43)	**<0.001**
Metastasis (positive/negative)	3	564	83 < 0.01	RE	4.66(1.13, 19.24)	**0.034**
Tumor stage (III+IV/I+II)	15	1778	70 0	RE	4.28(2.76, 6.63)	**<0.001**
Vascular invasion (positive/negative)	6	983	92 0	RE	1.55(0.51, 4.78)	0.441

TNM stages are based on tumor-node-metastasis classification advocated by International Union against Cancer.

LN metastasis, lymph node metastasis; OR, odds ratio; CI, confidence interval; FE, fixed-effect model; RE, random-effect model.

Bold fonts are used for all tables when the P value is less than 0.05.

### Associations of MTA1 Over-Expression With Survival

**Figure 3:**
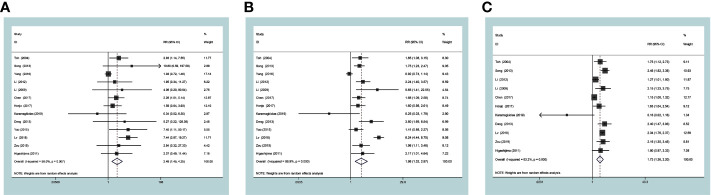
Forest plots of risk ratios for associations of metastasis-associated protein 1 (MTA1) with 1-year overall survival (OS) **(A)**, 3-yearOS **(B)** and 5-yearOS **(C)** in the gastrointestinal cancer (GIC) patients.

Survival datum was extracted from Kaplan-Meier survival curves by the Engauge Digitizer software. In the current study, the expression level of MTA1 was significantly associated with OS in ESCC, GC, CRC and GIC cases (**Table 2**). GIC patients with positive expression of MTA1 all showed worse OS; indeed, MTA1 was significantly associated with 1- (RR=2.48 [1.45–4.25], *P*=0.001, **Figure 3A**), 3- (RR=1.66 [1.30–2.11], *P*<0.001, **Figure 3B**) and 5-year (RR=1.73 [1.37–2.20], *P*<0.001, **Figure 3C**) OS.

**Table 2:** Meta-analysis of a putative association between OS and metastasis-associated protein 1 (MTA1) expression in gastrointestinal cancer.

Tumor type	OS	Number of studies	Number of patients	Heterogeneity	Model	RR(95%CI)	*P* value
				*I* ^2^ (%)*P* value			
Gastrointestinal cancers	1-year OS	13	1,766	56 0.01	RE	2.48(1.45, 4.25)	**0.001**
	3-year OS	13	1,766	70 <0.01	RE	1.66(1.30, 2.11)	**<0.001**
	5-year OS	11	1,508	83 <0.01	RE	1.73(1.37, 2.20)	**<0.001**
Esophageal cancer	1-year OS	8	983	33 0.17	FE	1.48 (1.11, 1.95)	**0.007**
	3-year OS	8	983	76<0.01	RE	1.60(1.12, 2.28)	**0.009**
	5-year OS	7	786	82<0.01	RE	1.55(1.14, 2.11)	**0.006**
Stomach cancer	1-year OS	3	608	0 0.98	FE	7.30(3.14, 16.99)	**<0.001**
	3-year OS	3	608	92<0.01	RE	3.23(1.12, 9.34)	**0.030**
	5-year OS	2	547	0 0.53	FE	2.08(1.80, 2.41)	**<0.001**
Colorectum cancer	1-year OS	2	175	0 0.88	FE	2.56(0.71, 9.25)	0.152
	3-year OS	2	175	0 0.84	FE	2.04(1.29, 3.22)	**0.002**
	5-year OS	2	175	0 0.65	FE	2.01(1.39, 2.92)	**<0.001**

OS, overall survival; RR, risk ratio; CI, confidence interval; FE, fixed-effect model; RE, random-effect model.

Bold fonts are used for all tables when the P value is less than 0.05.

**Figure 4:**
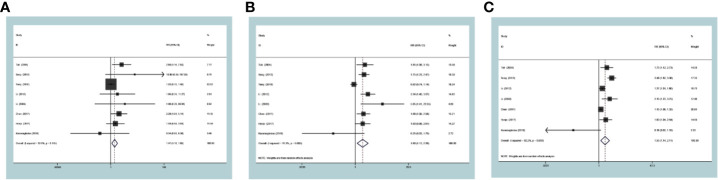
Forest plots of risk ratios for associations of metastasis-associated protein 1 (MTA1) with 1-year overall survival (OS) **(A)**, 3-year OS **(B)**, and 5-year OS **(C)** in the esophageal squamous cell carcinoma (ESCC) subgroup patients.

**Figure 5:**
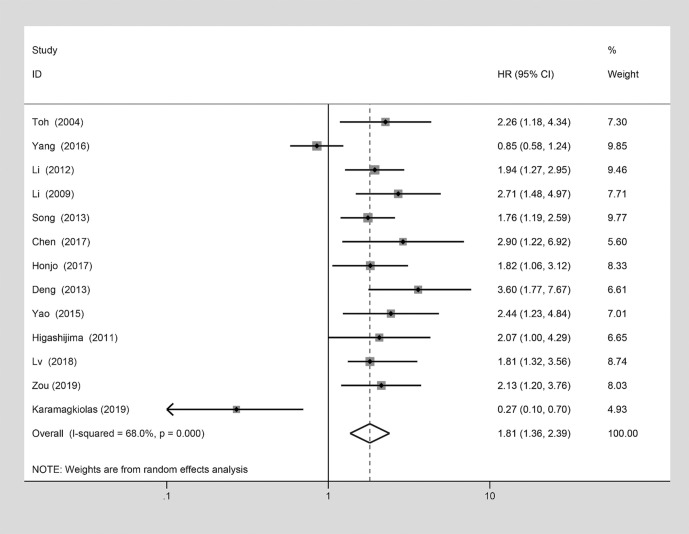
Forest plot of the association between metastasis-associated protein 1 (MTA1) over-expression and the overall survival (OS) which analyzed by the pooled HRs and their corresponded 95% conﬁdence intervals (CIs) from each eligible studies.

In the ESCC subgroup, MTA1 was also associated with 1- (RR=1.48 [1.11–1.95], *P*=0.007, **Figure 4A**), 3- (RR=1.60 [1.12–2.28], *P*=0.009, **Figure 4B**) and 5-year (RR=1.55 [1.14–2.11], *P=*0.006, **Figure 4C**) OS. In the GC subgroup, MTA1 was also associated with 1- (RR=7.30 [3.14–16.99], *P*<0.001, **Table 2**), 3-(RR=3.23 [1.12–9.34], *P*=0.03, **Table 2**) and 5-year (RR=2.08 [1.80–2.41], *P*<0.001, **Table 2**) OS. In the CRC subgroup, MTA1 was also associated with 3- (RR=2.037 [1.29–3.22], *P*=0.002, **Table 2**) and 5-year (RR=2.01 [1.39–2.92], *P*<0.001, **Table 2**) OS.

A meta-analysis was considered to explore the potential correlation between MTA1 and the OS, and it was analyzed by the pooled HRs and their corresponded 95% CIs from each eligible study. The results of pooled HRs and 95% CIs were displayed with details in **Table 3**. Among the 27 eligible studies, the terrible prognosis of GIC cases was showed in the pooled HR estimate (HR=1.81 [1.36–2.39], *P*<0.001, **Figure 5**). A significant association was observed in univariate analysis (HR=1.82 [1.35–2.45], *P*<0.001, **Table 3**), but not in the multivariate analysis (HR=1.89 [0.98–3.64], *P*=0.059, **Table 3**). Moreover, the relationship between MTA1 and OS vary in the different cancer types subgroup. As displayed in **Table 3**, MTA1 was related to prognosis of ESCC (HR=1.56 [1.04–2.33], *P*=0.03, **Table 3**), GC (HR=2.30 [1.61–3.27], *P*<0.001, **Table 3**) and CRC patients (HR=2.11 [1.34–3.30], *P*=0.001, **Table 3**). A significant association was observed in the more than 100 patients subgroup (HR=1.83 [1.35–2.47], *P*<0.001, **Table 3**), but not in the less than 100 patients subgroup.

**Table 3:** A meta-analysis of the pooled HRs to investigate the association between MTA1 expression and OS in patients with gastrointestinal cancer.

Categories	Subgroup	Studies	No of patients	HR	95% CI	*I* ^2^(%)	*P* value
Overall survival	Overall	13	1,800	1.81	(1.36, 2.39)	68	**<0.001**
Analysis of variable	Multivariate	4	834	1.89	(0.98, 3.64)	84	0.059
	Univariate	9	966	1.82	(1.35, 2.45)	54	**<0.001**
Cancer type	Esophagus	8	1,017	1.56	(1.04, 2.33)	77	**0.030**
	Stomach	3	608	2.30	(1.01, 3.27)	15	**<0.001**
	Colorectum	2	175	2.11	(1.34, 3.30)	0	**0.001**
Sample size	<100	5	364	1.64	(0.84, 3.20)	77	0.147
	>100	8	1,436	1.83	(1.35, 2.47)	64	**<0.001**

Bold fonts are used for all tables when the P value is less than 0.05.

### Subgroup Analysis

To further assess the correlation between the expression of MTA1 and prognosis of GIC patients, the selected studies were rigorously divided into subgroups based on language and tumor types. In these subgroups, MTA1 expression was meticulously assessed by different language (SCI articles and CNKI articles) groups and patients with ESCC, CRC and GC (**Supplementary Table 3**).

Among the articles in SCI and CNKI subgroups, the positive expression of MTA1 was close to clinical parameters. In the SCI articles subgroup, over-expression of MTA1 was tightly related to tumor size (OR=1.87 [1.10–3.18], *P*=0.021, **Supplementary Table 3**), depth of tumor invasion (OR=2.95 [2.36–3.68], *P<*0.001, **Supplementary Table 3**), lymphatic metastasis (OR=2.59 [1.57–4.27], *P*<0.001, **Supplementary Table 3**), and TNM stage (OR=4.14 [2.10–8.18], *P*<0.001, **Supplementary Table 3**), vascular invasion (OR=3.44 [1.84–6.42], *P<*0.001, **Supplementary Table 3**). A similar conclusion can be seen in the CNKI articles subgroup.

In the ESCC subgroup, higher expression of MTA1 conferred a higher risk of depth of invasion (OR=2.96 [2.28–3.84], *P*<0.001, **Supplementary Table 3**), increased odds of lymphatic metastasis (OR=2.79 [1.82–4.27], *P*<0.001, **Supplementary Table 3**) and advanced tumor stage (OR=3.30 [2.42–4.51], *P*<0.001, **Supplementary Table 3**). However, the expression of MTA1 showed no association with the remaining clinical parameters.

The expression of MTA1 was also close to clinical metastatic variables in patients with GC. MTA1 showed close associations with tumor tissue differentiation (OR=2.37 [1.21–4.64], *P*=0.012, **Supplementary Table 3**), depth of invasion (OR=2.84 [1.25–6.44], *P*=0.013, **Supplementary Table 3**), lymphatic metastasis (OR=5.80 [3.05–11.01], *P*<0.001, **Supplementary Table 3**), distant metastasis (OR=9.40 [5.06–17.46], *P*<0.001, **Supplementary Table 3**) and TNM stage (OR=14.69 [9.06–23.84], *P*<0.001, **Supplementary Table 3**) in GC.

Moreover, MTA1 expression was significantly related to metastasis-related clinical parameters in CRC patients. Indeed, high MTA1 expression always resulted in poor differentiation (OR=3.05[1.84–5.07, *P<*0.001, **Supplementary Table 3**), deep invasion (OR=2.25[1.04–4.89, *P*=0.04, **Supplementary Table 3**), increased odds of lymphatic metastasis (OR=2.51[1.57–4.02, *P*<0.001, **Supplementary Table 3**), and advanced tumor stage (OR=2.91[1.87–4.53, *P*<0.001, **Supplementary Table 3**).

### Sensibility Analysis

**Figure 6:**
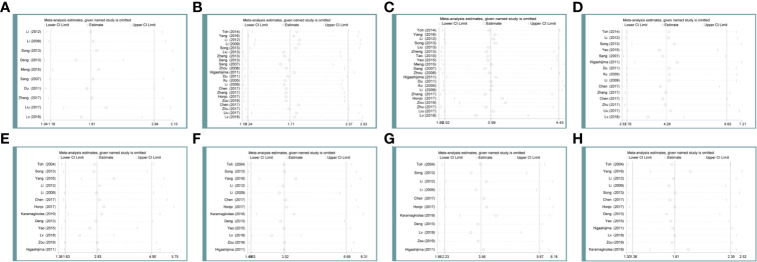
Sensibility analysis of metastasis-associated protein 1 (MTA1) expression with tumor size **(A)**, differentiation **(B)**, lymph node metastasis **(C)**, TNM stage **(D)**, 1-year **(E)** overall survival (OS), 3-year **(F)** OS, 5-year **(G)** OS, HR **(H)** in the gastrointestinal cancer (GIC) patients.

In the main studies which held heterogeneity, sensitivity analysis was used to determine whether the conclusions were stable. After deleting any of the studies, there was no significant change in the combined indicators of the remaining studies. It can be concluded that associations of MTA1 expression with tumor size (**Figure 6A**), differentiation (**Figure 6B**), lymph node metastasis (**Figure 6C**), TNM stage (**Figure 6D**), 1- (**Figure 6E**), 3- (**Figure 6F**), 5-year (**Figure 6G**) OS, HR (**Figure 6H**) are stable.

### Publication Bias

Both of the *Begg’s Rank Correlation* test and *Egger’s Weighted Regression* method were simultaneously applied to statistically assess publication bias. The *Begg’s* (*P*=0.980) and *Egger’s* (*P*=0.796) test gave out clear evidence of publication bias. These above results showed the credibility of the findings reported in this meta-analysis.

### MTA1 Expression and Prognosis in Gastric Cancer Patients Through Immunohistochemistry

**Figure 7:**
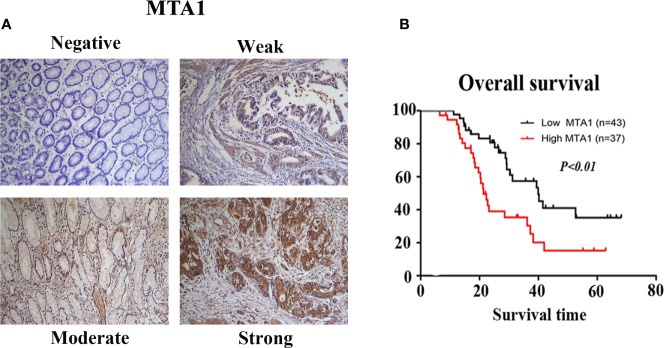
The results of immunohistochemistry (IHC) staining confirmed the expression of metastasis-associated protein 1 (MAT1) is correlated with the TNM stage **(A)** and overall survival OS **(B)** of gastrointestinal cancer (GC) patients.

The potential relationships between the expression level of MTA1 and clinicopathological parameters were displayed in **Table 4**. In this paper, we found that the expression level of MTA1 was significantly higher in GC samples than it in adjacent non-tumor samples through analyzing 80 paired IHC-treated paraffin-embedded sections of GC samples. Here no statistical difference of MTA1 expression was found in some clinical parameters, such as gender (*P*=0.216, **Table 4**), age (*P*=0.861, **Table 4**) and tissue differentiation grade (*P*=0.379, **Table 4**). However, as **Table 4** showed, higher expression of MTA1 accompanied with larger tumor size (*P*=0.008, **Table 4**), higher rate of lymph node metastasis (*P*=0.007, **Table 4**), and higher rate of distant metastasis (*P*=0.023, **Table 4**). According to the IHC staining results, increased expression of MTA1 was accompanied by the worse tumor TNM stage (**Figure 7**). Furthermore, Kaplan-Meier analysis of 80 GC samples, grouped by the MTA1 expression, which measured by IHC, showed that higher expression of MTA1 with worse prognosis in GC patients (*P*<0.01, **Figure 7**).

**Table 4:** The correlations between MTA1 protein over-expression and clinicopathological parameters in GC.

Parameters	Cases	MTA1 expression		*X^2^*	*P* value
		High	Low		
Gender				1.528	0.216
Male	46	24	22		
Female	34	13	21		
Age (years)				0.030	0.861
≥60	62	29	33		
<60	18	8	10		
Tumor size (cm)				7.011	**0.008**
≥5	37	23	14		
<5	43	14	29		
Differentiation				0.775	0.379
Well + moderate	41	17	24		
Poor	39	20	19		
LN metastasis				7.242	**0.007**
Yes	55	31	24		
No	25	6	19		
Distant metastasis				5.144	**0.023**
Yes	17	12	5		
No	63	25	38		

GC, gastric cancer; LN metastasis, lymph node metastasis. Bold fonts are used for all tables when the P value is less than 0.05.

### Univariate and Multivariate Analysis of Overall Survival

The potential interaction, which determined GC prognosis, between the expression level of MTA1 and clinical factors was assessed with the help of univariate and multivariate analysis, respectively. In the results of univariate analysis, tumor size (HR = 1.922 [1.119–3.548], *P*= 0.019, **Table 5**), lymph node metastasis (HR = 1.927 [1.054–3.523], *P* = 0.033, **Table 5**), distant metastasis (HR = 5.572 [2.660–11.671], *P*< 0.001, **Table 5**) and MTA1 expression (HR = 2.778 [1.550–4.977], *P* = 0.001, **Table 5**) were associated with OS in GC patients. The multivariate Cox proportional hazard model showed that distance metastasis (HR = 4.077 [1.835–9.058], *P* = 0.001, **Table 5**) and MTA1 expression (HR = 2.061 [1.066–3.986], *P* = 0.032, **Table 5**) were tightly close to the OS in GC patients, which suggested that the expression of MTA1 might be an independent prognostic factor for gastric cancer.

**Table 5:** Univariate and multivariate analysis of clinicopathological variables and MTA1 expression associated with overall survival.

Parameters	Univariate analysis		Multivariate analysis	
	HR (95% CI)	*P*-value	HR (95% CI)	*P*-value
**Gender**	0.807(0.459–1.420)	0.458		
**Age (years)**	1.403(0.699–2.817)	0.341		
**Tumor size (cm)**	1.992(1.119–3.548)	**0.019**	1.172(0.588–2.334)	0.652
**Differentiation**	1.183(0.668–2.093)	0.564		
**LN metastasis**	1.927(1.054–3.523)	**0.033**	1.291(0.659–2.526)	0.456
**Distant metastasis**	5.572(2.660–11.671)	**<0.001**	4.077(1.835–9.058)	**0.001**
**MTA1 expression**	2.778(1.550–4.977)	**0.001**	2.061(1.066–3.986)	**0.032**

Bold fonts are used for all tables when the P value is less than 0.05.

## Discussion

The depth of tumor invasion (T), lymphatic metastasis (N), distant metastasis (M) stages and TNM stage are wildly considered the pivotal important prognostic index of GIC, including GC (39). Although patients undergo the complete surgical resection whose postoperative pathologic stage of GIC are same, clinical evidence demonstrates that survival is different among them, which indicates that the current tumor evaluation system misses the accurate prediction of prognosis. Prognosis of GIC patients is usually predicted by TNM staging in the clinic, although low sensitivity is sometimes encountered. To our knowledge that different digestive tract cancers show a high risk of tumor recurrence and metastasis. And even accepted with complete surgical resection or targeted therapy, lots of GIC patients still meet death events caused by local recurrence and/or distant metastasis. So, finding new predictors with good prognostic value that can timely find out the patients who may hold poor survival is urgently needed.

The members of the MTA family hold pivotal roles in both pathological and physiological processes, especially in cancer progression (such as invasion and distant metastasis), and their role as master regulators has been reported. Proteins of the MTA family are involved in metastasis regulation and comprise MTA1, MTA2, and MTA3, which are found in different isoforms such as MTA1, MTA1s, MTA2, MTA3, MTA3L, and MTA-ZG29p (40, 41). The primary founding of MTA1 was in rat metastatic tumors, and it is considered as a metastasis-associated gene (42). Since then, abnormal expression level of MTA1 has been wildly reported in various types of cancers. Nevertheless, its biomolecular roles in the carcinogenic process remain unclear until it is identified as an integral component of the NuRD complex (43). Luo et al. (44) performed a meta-analysis to further carry out the functions of MTA1 in solid tumors and pointed out that the expression level of MTA1 was tightly close to the prognosis. In addition, Ning et al. (45) demonstrated that the expression level of MTA1 is close to tumor invasion and lymph node metastasis in GIC. However, the role of MTA1 in the prediction of prognosis is not yet well understood in GIC. Indeed, several studies have implied that MTA1 without worth in the evaluation of OS in cancers, such as in esophageal squamous cell carcinoma and breast cancer (14, 46).

In this study, IHC results showed that tumor size, lymph node metastasis and distant metastasis were associated with MTA1 expression in GC patients. The K-M curve showed that GC patients with high expression of MTA1 had lower OS. The multivariate Cox proportional hazard model showed that MTA1 expression may be an independent prognostic factor in gastric cancer. In addition to gastric cancer, we also obtained similar results in GIC in meta-analysis. However, there was no statistical correlation between differentiation and MTA1 expression in the results of IHC, contrary to the conclusion reached by meta-analysis. The contradiction between the two conclusions may be caused by too few IHC samples.

From a clinical perspective, the expression level of MTA1 holds close associations with depth of invasion, lymph node metastasis, distant metastasis and TNM stage, which are separate and independent but internally linked parameters. Tumor tissues with MTA1 expression shows increased invasion, which traverses the lymphatic network under the mucosa, with a higher possibility of vascular invasion; finally, the tumor stage becomes advanced, resulting in reduced OS. Furthermore, the following molecular mechanisms are obtained for MTA1. As shown in previous studies, MTA1 may act as a transcriptional regulator; in fact, MTA1 mediates transcription repression through interacting with NuRD which facilitates the association between repressor molecules and chromatin (41, 47, 48). For example, MTA1 can directly bind to HDAC1 (49); both of them are the main components of the NuRD complex, by which it contains histone deacetylase, and acts as a pivotal role in histone deacetylation, chromatin alteration and transcriptional control. Toh et al. (13) pointed out that MTA1 is close to H4 histone deacetylase activity in ESCC. Of note, tumor suppressor genes, including p53, p21, and the Bcl-2 family of proteins, are regulated by histone acetylation (50, 51).

In a meta-analysis conducted in 2017 (52), we have preliminarily confirmed that the expression of MTA1 is statistically related to clinicopathological features and survival rates in patients with GIC. However, only 13 articles were included in previous studies, which may lead to errors in the results. On the basis of the original study, 14 new articles (27 articles in total) were included in this study, and the original results were updated. We found that the tumor size, differentiation, distant metastasis and high expression of MTA1 in GIC patients had no statistical correlation in the previous study, but the updated data showed all of them had a statistical correlation. Meantime, we found that vascular invasion and high expression of MTA1 in GIC patients had a statistical correlation in the previous study, but the updated data showed no statistical correlation between the two. Previous studies and current studies have shown that high MTA1 expression indicates low OS in patients with GIC. In this study, we also concluded that high expression of MTA1 indicates low HR in patients with GIC, which provides the latest evidence for the above conclusion.

The limitations of this meta-analysis should be noted (1): some eligible non-English and non-Chinese publications may have been excluded (2); IHC assessments of MTA1 remain discordant. Meanwhile, this study has several advantages (1): it is the first available study that applies meta-analysis to evaluate associations between MTA1 and HRs in GIC (2); we updated the data from previous studies and draw more reliable conclusions (3). we detected the expression of MTA1 in gastric cancer and verified the prognostic value of MTA1.

In conclusion, the expression level of MTA1 is obviously associated with clinical and pathological parameters and OS in GIC patients. And, it may make contributions in predicting aggressive tumor behavior and poor prognosis as an independent factor. The results of this study also imply that MTA1 is a potential target for anticancer therapy. Further research is required to unveil the mechanisms underlying MTA1 function.

## Data Availability Statement

All datasets generated for this study are included in the article/**Supplementary Material**.

## Author Contributions

GC and PL conceptualized the study. BC, GC, PL, and WC contributed to the methodology. GC, WC, RD, and MC were in charge of the software. PL, WC, GC, and MC conducted the formal analysis. GC, BC, and SC conducted the investigation. GC, RD, XX, WC,and MC performed the data curation. BC and MX were in charge of the project administration. GC wrote and prepared the original draft. SC, BC, MX wrote, reviewed, and edited the manuscript. GC and XX conducted the visualization. BC and MX acquired the funding. All authors contributed to the article and approved the submitted version.

## Funding

This work was supported by the Key Research and Development Plan Projects of Anhui Province (Project No. 201904a07020045).

## Conflict of Interest

The authors declare that the research was conducted in the absence of any commercial or financial relationships that could be construed as a potential conflict of interest.
