# Supplementary material for: Expression and Prognostic Significance of Metastasis-Associated Protein 1 in Gastrointestinal Cancer
Source: Front Oncol. 2020 Dec 21;10:542330. doi: 10.3389/fonc.2020.542330 (PMC7780747; doi:10.3389/fonc.2020.542330)
Supplement: Supplementary file 1 [file Table_1.docx]

| Studies | Tumor location | Number of Patients  (positive vs. negative) | Sex (male/female)  MTA1(+) MTA1(-) | Age (<60/>60)  MTA1(+) MTA1(-) | Tumor size (<5cm/>5cm)  MTA1(+) MTA1(-) | Differentiation (well/poor)  MTA1(+) MTA1(-) | Depth of invasion  (T1+T2/T3+T4)  MTA1(+) MTA1(-) | LN metastasis (positive/negative)  MTA1(+) MTA1(-) | Distant metastasis (positive/negative)  MTA1(+) MTA1(-) | Tumor stage (early/anvanced)  MTA1(+) MTA1(-) | Vascular invasion (positive/negative)  MTA1(+) MTA1(-) | Quality  score |
| --- | --- | --- | --- | --- | --- | --- | --- | --- | --- | --- | --- | --- |
| Toh 2004 | Esophagus | 70(30vs.40) | 24/6 37/3 | NA | NA | 22/8 26/14 | 10/20 30/10 | 21/9 15/25 | NA | 13/17 28/12 | 15/15 11/29 | 9 |
| Yang 2016 | Esophagus | 197(83vs.114) | 63/20 85/29 | 38/45 55/59 | NA | 65/19 93/21 | 28/55 58/56 | 45/38 43/71 | NA | NA | NA | 8 |
| Li 2012 | Esophagus | 131(57vs.74) | 40/17 55/19 | NA | 36/21 57/17 | 45/12 50/24 | 18/39 38/36 | 37/20 33/41 | NA | 26/31 55/19 | NA | 8 |
| Li 2009 | Esophagus | 90(40vs.50) | 28/12 34/16 | NA | 24/16 35/15 | 30/10 31/19 | 11/29 27/23 | NA | NA | NA | NA | 8 |
| Song 2013 | Esophagus | 174(79vs.95) | 60/19 70/25 | NA | 48/31 63/32 | 63/16 76/19 | 18/61 41/54 | 48/31 43/52 | NA | 41/38 64/31 | NA | 5 |
| Liu 2013 | Esophagus | 81(59vs.22) | 50/9 15/7 | NA | NA | 27/31 18/5 | 17/42 16/6 | 44/15 9/13 | NA | NA | NA | 5 |
| Zheng 2013 | Esophagus | 44(27vs.17) | 16/11 11/6 | 12/15 10/7 | NA | 15/12 15/2 | NA | 20/7 5/12 | NA | NA | NA | 6 |
| Tao 2010 | Esophagus | 64(42vs.22) | 24/18 15/7 | 20/22 10/12 | NA | NA* | NA | 25/17 5/17 | NA | NA | NA | 6 |
| Chen 2017 | Esophagus | 144(60vs.84) | 25/35 58/26 | 19/41 34/50 | NA | 46/14 58/26 | 26/34 62/22 | NA | NA | 26/34 70/14 | NA | 7 |
| Zhang 2017 | Esophagus | 84(59vs.25) | 35/24 14/11 | 34/25 17/8 | 30/29 16/9 | 30/29 19/6 | 40/19 21/4 | 41/18 6/19 | NA | 36/23 22/3 | NA | 6 |
| Zhang 2018 | Esophagus | 48(35vs.13) | NA | NA | NA | NA | 19/16 12/1 | NA | NA | NA | NA | 4 |
| Zhu 2017 | Esophagus | 96(71vs.25) | 44/27 13/12 | NA | NA | 46/25 20/5 | NA | 29/42 2/23 | NA | 36/35 21/4 | NA | 6 |
| Honjo 2017 | Esophagus | 142(82vs.60) | 7/75 9/51 | NA | NA | 55/27 51/9 | NA | 31/51 28/32 | NA | NA | 13/69 24/36 | 7 |
| Karamagkiolas 2019 | Esophagus | 69(20vs.49) | NA | NA | NA | NA | NA | NA | NA | NA | NA | 7 |
| Liu 2017 | Esophagus | 107(54vs.53) | 45/9 41/12 | 21/33 22/31 | 40/14 32/21 | 46/8 43/10 | 13/41 24/29 | 37/17 26/27 | NA | 24/30 36/17 | NA | 7 |
| Deng 2013 | Stomach | 111(40vs.71) | 30/10 57/14 | NA | 23/17 67/4 | 17/23 43/28 | 31/9 58/13 | NA | NA | NA | NA | 6 |
| Yao 2015 | Stomach | 61(47vs.14) | 16/31 7/7 | NA | NA | NA | NA | 37/10 6/8 | NA | 7/40 10/4 | NA | 8 |
| Meng 2015 | Stomach | 160(70vs.90) | 47/23 67/23 | NA | 47/23 62/28 | NA | 14/56 35/55 | 59/11 58/32 | NA | NA | 31/39 26/64 | 8 |
| Sang 2007 | Stomach | 54(25vs.29) | 18/7 19/10 | 12/13 12/17 | 16/9 21/8 | 11/14 25/4 | NA | 18/7 7/22 | 10/15 2/27 | 6/19 20/9 | NA | 6 |
| Zhou 2008 | Stomach | 67(45vs.22) | 27/18 13/9 | NA | NA | 21/24 16/6 | NA | 31/14 8/14 | NA | NA | NA | 5 |
| Lv 2018 | Stomach | 436(162/274) | 115/47 196/78 | NA | 67/95 189/85 | 46/116 95/179 | 26/136 140/134 | 147/15 123/151 | 49/113 12/262 | 16/146 178/96 | 49/113 12/262 | 6 |
| Higashijima 2011 | Colorectum | 74(38vs.36) | 25/13 20/16 | NA | NA | 37/1 33/3 | 14/24 21/15 | 22/16 17/19 | 15/23 12/24 | 12/26 12/24 | 21/17 12/24 | 6 |
| Du 2011 | Colorectum | 81(25vs.56) | 17/8 28/28 | 11/14 23/33 | 10/15 36/20 | 15/10 44/12 | 3/22 12/44 | 17/8 23/33 | NA | 8/17 33/23 | NA | 8 |
| Xu 2005 | Colorectum | 84(52vs.32) | NA | 29/23 18/14 | NA | 34/18 28/4 | NA | 29/23 10/22 | NA | 22/30 22/10 | NA | 5 |
| Li 2009 | Colorectum | 78(49vs.29) | 31/18 23/6 | NA | NA | 27/22 24/5 | NA | 34/15 12/17 | NA | 14/35 16/13 | NA | 4 |
| Chen 2017 | Colorectum | 104(88vs.16) | 69/19 10/6 | NA | NA | 64/24 16/0 | NA | NA | NA | 30/58 14/2 | NA | 4 |
| Zou 2019 | Colorectum | 101(51vs.50) | 31/20 35/15 | 10/41 16/34 | NA | 38/13 45/5 | NA | 21/30 33/17 | NA | NA | 27/24 37/13 | 6 |

**Supplementary table 1.** Clinicopathological parameters and quality scores of sutides comparing MTA1 positive gastrointestinal cancer with MTA1 negative gastrointestinal cancer

LN: lymph node; NA: not available; Red represents Chinese literatures

TNM stages are based on tumor-node-metastasis classification advocated by International Union against Cancer

Quality score: use the Newcastle-Ottawa scale (stars)
