# Supplementary material for: Expression and Prognostic Significance of Metastasis-Associated Protein 1 in Gastrointestinal Cancer
Source: Front Oncol. 2020 Dec 21;10:542330. doi: 10.3389/fonc.2020.542330 (PMC7780747; doi:10.3389/fonc.2020.542330)
Supplement: Supplementary file 2 [file Table_2.docx]

**Supplementary table 2.** IHC antibodies and assessment methods of MTA1 expression in the eligible studies.

| Study | Year | Tumor Type | Antibody | Antibody Concentration | The Positive-cell Scoring | Staining Intensity | IHC Assessment Method |
| --- | --- | --- | --- | --- | --- | --- | --- |
| Toh | 2004 | Esophageal | NA | NA | NA | Score: no staining (0); slight staining (+); moderate staining (++); intense staining (+++). | Scores were compared between the carcinoma tissues and the normal squamous epithelium contained in the same section. In all cases, the normal epithelial cells were scored (+), and the scores (++) and (+++) in the carcinoma tissues were defined as overexpression of MTA1 protein |
| Yang | 2016 | Esophageal | sc-9446, Santa Cruz Biochemistry | 1/100 | Positive: <5%, 0 points; 5-25%, 1 point; 26-50%, 2 points; 51-75%, 3 points; and >75%, 4 points. | Staining intensity: Minimal staining similar to the background, 0 points; lightly stained, more than the background and pale yellow, 1 point; moderately stained, markedly more than the background and a brown-yellow, 2 points; and clearly stained a dark brown-yellow or tan, 3 points. | Total score: number of positive cells x staining intensity. Total score ≥5 indicated a positive result, and <5 indicated  a negative result. |
| Li | 2012 | Esophageal | sc-9446, Santa Cruz Biotechnology | 1/100 | Positive: 0, 0–5%; 1,6–25%; 2, 26–50%; 3, 51–75%; 4, >75% | Staining intensity: 0, negative; 1, weak; 2, moderate; 3,strong | The final staining score was the sum of the scores of staining intensity and percentage of positive cells, ranging from 0 to 7. |
| Li | 2009 | Esophageal | sc-9446, Santa Cruz Biotechnology | 1/100 | NA | Staining intensity and proportion of the stained tumor nuclei as follows: score 0, no staining; (+), slight staining; (++), moderate staining;(+++), intense staining. | For all cases, the normal epithelial cells that were scored (+), and the cancer tissues that were scored (++) and (+++) were defied as overexpression of MTA1 protein |
| Song | 2013 | Esophageal | sc-9446, Santa Cruz Biochemistry | 1/100 | Positive: 0, 0–5%; 1, 6–25%; 2, 26–50%; 3, 51–75%; 4, >75% | Staining intensity: 0, negative staining; 1, weak staining; 2,moderate staining; 3, intense staining | The final staining score was the sum of the scores of staining intensity and percentage of positive cells: (-), 0 to 1; (+), 2 to 3;(++), 4 to 5; (+++), 6 to 7. |
| Liu | 2013 | Esophageal | NA | NA | NA | NA | The yellow staining of nucleus was regarded as positive cell, samples with 10% tumor cells were defined as positive. |
| Zheng | 2013 | Esophageal | Boster Biological Technology | NA | Positive:1, 0–25%; 2, 26–50%; 3, 51–75%; 4, >75% | Staining intensity: 0, negative; 1, weak; 2, moderate; 3,strong | The final staining score was the sum of the scores of staining intensity and percentage of positive cells: ≥1 was defied as positive expression |
| Tao | 2010 | Esophageal | Santa Cruz Biochemistry | 1/200 | Positive: 0, 0–10%; 1, 11–50%; 2, 51–75%; 3, >75% | Staining intensity: 0, negative; 1, weak; 2, moderate; 3,strong | The final staining score was the sum of the scores of staining intensity and percentage of positive cells: ≥2 was defied as positive expressio |
| Chen | 2017 | Esophagus | NA | NA | Positive: 0%, 0 points; 10%, 1 point; 10-50%, 2 points;>50%, 3 points. | Staining intensity: Minimal staining similar to the background, 0 points; lightly stained, more than the background and pale yellow, 1 point; moderately stained, markedly more than the background and a brown-yellow, points; and clearly stained a dark brown-yellow or tan, 3 points. | Total score: number of positive cells x staining intensity. Total score ≦4 indicated a negative result; and >4 and≦9 indicated a moderate positive result. |
| Zhang | 2017 | Esophagus | NA | NA | Positive: <10%, 0 points; 0-50%, 1 point; 51-75%, 2 points;>75%, 3 points. | Staining intensity: Minimal staining similar to the background, 0 points; lightly stained, more than the background and pale yellow, 1 point; moderately stained, markedly more than the background and a brown-yellow, 2 points; and clearly stained a dark brown-yellow or tan, 3 points. | Total score: number of positive cells + staining intensity. Total score ≥2 indicated a positive result, and <2 indicated a negative result. |
| Zhu | 2017 | Esophagus | NA | NA | Positive: <10%, 0 points; 10-49%, 1 point; 50-74%, 2 points;>74%, 3 points. | Staining intensity: Minimal staining similar to the background, 0 points; lightly stained, more than the background and pale yellow, 1 point; moderately stained, markedly more than the background and a brown-yellow, 2 points; and clearly stained a dark brown-yellow or tan, 3 points. | Total score: number of positive cells + staining intensity. Total score ≥2 indicated a positive result, and <2 indicated a negative result. |
| Honjo | 2017 | Esophagus | D40D1, Cell Signaling Technology | 1:300 | NA | NA | NA |
| Karamagkiolas | 2019 | Esophagus | antiMTA1 | 1:10 | a four-scale intensity scheme :0: negative; 1: weak, 2: moderate, and 3: high staining intensity. | The percentage of immunopositive cells :<30% were considered as weakly stained; 31% - 69% as moderately stained, and above 70% as strongly stained. | IHC staining score: 0 or 1 indicated a negative result ; IHC staining score: 2 or indicated a positive result |
| Liu | 2017 | Esophagus | sc - 17773 | 1:50 | positive cells: 0, ≤5%; 1, 6‑25%; 2, 26‑50%; 3, 51‑75%; 4, 76-100% | Staining intensity: 0, no signal; 1, pale yellow; 2, buffy; 3, brownish. | Total score: the positive cell proportion x the staining intensity: 0, 0-1 score; 1, 2-4 score; 2, 6-8 score; 3, 9-12 score; high expres-  sion, ≥2 points; low expression, <2 points (12,13). |
| Zhang | 2017 | Esophageal | NA | NA | Positive: 0, 0–10%; 1, 11–50%; 2, 51–75%; 3, >75% | Staining intensity: 0, negative; 1, weak; 2, moderate; 3,strong | The final staining score was the sum of the scores of staining intensity and percentage of positive cells: ≥3 was defied as positive expression |
| Deng | 2013 | Gastric | sc-9446, Santa Cruz Biochemistry | 1/100 | NA | The results were reported as follows: 0, no staining; +, slight staining; ++, moderate staining; +++, intense staining. | The cancer tissues scored as ++ and +++ were defined as exhibiting overexpression of MTA1 protein. |
| Yao | 2015 | Gastric | Santa Cruz Biochemistry | 1/500 | Positive: 0%, negative, 5%, weak positive; 5%–25%, intermediate; 25%–50%, moderate; 50%–100%, strong) | NA | The distribution of tumor cells in all experimental groups was determined as follows: 0%–5%is lower expression and 5%–100% is higher expression. |
| Meng | 2015 | Gastric | # 5647, Cell Signaling | 1/100 | <25%, 1; 25–50%, 2; >50%-<75%, 3; >75%, 4 scores | Staining intensity: negative ,0; weak, 1; moderate, 2; or strong, 3 scores | A staining index (values 0–12), >6 indicated a positive result. |
| Sang | 2007 | Gastric | sc-9446, Santa Cruz Biochemistry | NA | 0%, 0; <25%, 1; 25–50%, 2; 50% –75%, 3; >75%, 4 scores | Staining intensity: negative ,0 ; weak, 1;moderate, 2; or strong, 3 scores | The final staining score was the sum of the scores of staining intensity and percentage of positive cells: (-), 0; (+), 1-2; (++), 3-4; (+++), >4. |
| Zhou | 2008 | Gastric | Zsbio Biochemistry | 1/100 | Positive: 0, 0–10%; 1, 11–50%; 2, 51–75%; 3, >75% | Staining intensity: negative ,0 ; weak, 1;moderate, 2; or strong, 3 scores | The final staining score was the sum of the scores of staining intensity and percentage of positive cells: ≥2 was defied as positive expression |
| Lv | 2018 | Gastric | NA | NA | Positive:0,≤5%; 1, 6–25%; 2, 26–50%,;and 3, > 51%. | Staining intensity:0, none (−);1, weak (light yellow, +);2, moderate (yellow brown,++); and 3, strong (brown,+++). | Total score: the positive cell proportion x the staining intensity: ≥4 was defined as positive ,≤3 was defined as negetive. |
| Higashijima | 2011 | Colorectal | sc-17773, Santa Cruz Biochemistry | 1/10 | NA | NA | Regarding the assessment of staining, the tumor was defined as exhibiting positive staining when >10% nuclear staining of the protein was noted in the tumor tissue. |
| Du | 2011 | Colorectal | sc-9446, Santa Cruz Biochemistry | NA | Samples with 10% tumor cells were defined as positive. | Staining intensity: 0 (no staining), 1 (weak staining), 2 (moderate staining), and 3 (strong staining) | Tumors with a score > 2 (moderate and strong expression) showed a high expression level of MTA1. |
| Xu | 2005 | Colorectal | sc-9446, Santa Cruz Biochemistry | 1/200 | Positive: 0, 0–10%; 1, 11–50%; 2, 51–75%; 3, >75% | Staining intensity:: negative (score = 0), weak (score = 1), moderate (score = 2), or strong (score = 3) | Tumors with scores of more than 2 were considered to show MTA1 overexpression. |
| Li | 2009 | Colorectal | Maxim Biotechnology | NA | NA | NA | The yellow staining of nucleus was regarded as positive expression. |
| Chen | 2017 | Colorectal | 1C3, Santa Cruz Biochemistry | 1/100 | Positive: 0, 0%; 1, 10–30%; 1, 31–60%; 3, >60% | Staining intensity: negative ,0; weak, 1; moderate, 2; or strong, 3 scores | Tumors with scores of more than 3 were considered to show MTA1 overexpression. |
| Zou | 2019 | Colorectal | Cell signalling technology，CST5674 | 1/50 | Positive: <10%, 0 points; 10%-<50%, 1 point; 50-75%, 2 points;>75%, 3 points. | Staining intensity: Minimal staining similar to the background, 0 points; lightly stained, more than the background, 1 point ;moderately stained, markedly more than the background, 2 points; and clearly stained a dark brown-yellow or tan, 3 points. | Total score(0-9): number of positive cells x staining intensity. Total score 0 indicated a negative result; 1-3 indicated a weakly positive result; 4-6 indicated a moderately positive result; 7-9 indicated a strongly positive result. |

NA: not availabl
