# Supplementary material for: Expression and Prognostic Significance of Metastasis-Associated Protein 1 in Gastrointestinal Cancer
Source: Front Oncol. 2020 Dec 21;10:542330. doi: 10.3389/fonc.2020.542330 (PMC7780747; doi:10.3389/fonc.2020.542330)
Supplement: Supplementary file 3 [file Table_3.docx]

**Supplementary table 3.** Subgroup analysis according to tumor location: Meta-analysis of the association between clinicopathological parameters and MTA1 expression

| Subgroup type | Parameters | Number of studies | Number of patients | Heterogeneity | Model | OR(95%CI) | *P* value |
| --- | --- | --- | --- | --- | --- | --- | --- |
|  |  |  |  | *I*^2^ (%) *P* value |  |  |  |
| Esophageal cancer | Sex (male/female) | 13 | 1424 | 35 0.10 | FE | 0.88(0.69, 1.12) | 0.281 |
|  | Age (>60/<60) | 6 | 640 | 0 0.93 | FE | 0.81(0.582, 1.11) | 0.190 |
|  | Tumor size (>5cm/<5cm) | 5 | 586 | 35 0.19 | FE | 1.27(0.90, 1.80) | 0.174 |
|  | Differentiation (poor/well) | 12 | 1360 | 60 <0.01 | RE | 1.28(0.84, 2.00) | 0.251 |
|  | Depth of invasion (T3+T4/T1+T2) | 10 | 1126 | 0 0.55 | FE | 2.96(2.28, 3.84) | **<0.001** |
|  | LN metastasis (positive/negative) | 11 | 1190 | 62 <0.01 | RE | 2.79(1.82, 4.27) | **<0.001** |
|  | Tumor stage (III+IV/I+II) | 7 | 806 | 17 0.30 | FE | 3.30(2.42, 4.51) | **<0.001** |
|  | Vascular invasion (positive/negative) | 2 | 212 | 92 <0.01 | RE | 0.84(0.10, 7.53) | 0.880 |
| Gastric cancer | Sex (male/female) | 6 | 889 | 0 0.83 | FE | 0.88(0.65, 1.19) | 0.399 |
|  | Age (>60/<60) | 1 | 54 | - | - | - |  |
|  | Tumor size (>5cm/<5cm) | 4 | 761 | 80 <0.01 | RE | 2.65(1.12, 6.26) | **0.027** |
|  | Differentiation (poor/well) | 4 | 668 | 61 0.05 | RE | 2.37(1.21, 4.64) | **0.012** |
|  | Depth of invasion (T3+T4/T1+T2) | 3 | 707 | 76 0.02 | RE | 2.84(1.25, 6.44) | **0.013** |
|  | LN metastasis (positive/negative) | 5 | 778 | 58 0.05 | RE | 5.80(3.05, 11.00) | **<0.001** |
|  | Metastasis (positive/negative) | 2 | 490 | 0 1.00 | FE | 9.40(5.06, 17.46) | **<0.001** |
|  | Tumor stage (III+IV/I+II) | 3 | 551 | 0 0.44 | FE | 14.69(9.06, 23.84) | **<0.001** |
|  | Vascular invasion (positive/negative) | 2 | 596 | 91 <0.01 | RE | 4.30(0.92, 20.20) | 0.065 |
| Colorectal cancer | Sex (male/female) | 5 | 438 | 48 0.10 | FE | 1.09(0.72, 1.67) | 0.679 |
|  | Age (>60/<60) | 3 | 266 | 0 0.46 | FE | 0.82(0.49, 1.39) | 0.469 |
|  | Tumor size (>5cm/<5cm) | 1 | 81 | - | - | - | - |
|  | Differentiation (poor/well) | 6 | 522 | 6 0.38 | FE | 3.05(1.84, 5.07) | **<0.001** |
|  | Depth of invasion (T3+T4/T1+T2) | 2 | 155 | 0 0.83 | FE | 2.25(1.04, 4.89) | **0.040** |
|  | LN metastasis (positive/negative) | 5 | 418 | 78 <0.01 | RE | 1.68(0.70, 4.02) | 0.243 |
|  | Metastasis (positive/negative) | 1 | 74 | - | - | - | - |
|  | Tumor stage (III+IV/I+II) | 5 | 421 | 49 0.10 | FE | 2.91(1.87, 4.53) | **<0.001** |
|  | Vascular invasion (positive/negative) | 2 | 175 | 88 <0.01 | RE | 0.98(0.16, 5.87) | 0.978 |
